# Alteration in the Wnt microenvironment directly regulates molecular events leading to pulmonary senescence

**DOI:** 10.1111/acel.12240

**Published:** 2014-07-01

**Authors:** Tamas Kovacs, Veronika Csongei, Diana Feller, David Ernszt, Gabor Smuk, Veronika Sarosi, Laszlo Jakab, Krisztian Kvell, Domokos Bartis, Judit E Pongracz

**Affiliations:** 1 Medical School, Department of Pharmaceutical Biotechnology, University of PécsPécs, Hungary; 2 János Szentágothai Research Centre, University of PécsPécs, Hungary; 3 Medical School, Department of Pathology, University of PécsPécs, Hungary; 4 Medical School, Department of Pulmonology, University of PécsPécs, Hungary; 5 Medical School, Department of Surgery, University of PécsPécs, Hungary; 6 Department of Clinical Respiratory Sciences, Centre for Translational Inflammation Research, University of Birmingham Research Laboratories, Queen Elizabeth HospitalBirmingham, UK

**Keywords:** molecular biology of aging, pulmonary senescence, Wnt microenvironment

## Abstract

In the aging lung, the lung capacity decreases even in the absence of diseases. The progenitor cells of the distal lung, the alveolar type II cells (ATII), are essential for the repair of the gas-exchange surface. Surfactant protein production and survival of ATII cells are supported by lipofibroblasts that are peroxisome proliferator-activated receptor gamma (PPARγ)-dependent special cell type of the pulmonary tissue. PPARγ levels are directly regulated by Wnt molecules; therefore, changes in the Wnt microenvironment have close control over maintenance of the distal lung. The pulmonary aging process is associated with airspace enlargement, decrease in the distal epithelial cell compartment and infiltration of inflammatory cells. qRT–PCR analysis of purified epithelial and nonepithelial cells revealed that lipofibroblast differentiation marker parathyroid hormone-related protein receptor (PTHrPR) and PPARγ are reduced and that PPARγ reduction is regulated by Wnt4 via a β-catenin-dependent mechanism. Using a human *in vitro* 3D lung tissue model, a link was established between increased PPARγ and pro-surfactant protein C (pro-SPC) expression in pulmonary epithelial cells. In the senile lung, both Wnt4 and Wnt5a levels increase and both Wnt-s increase myofibroblast-like differentiation. Alteration of the Wnt microenvironment plays a significant role in pulmonary aging. Diminished lipo- and increased myofibroblast-like differentiation are directly regulated by specific Wnt-s, which process also controls surfactant production and pulmonary repair mechanisms.

## Introduction

In the aging lung, the total tissue mass decreases along with the number of capillaries. Formation of new alveoli is also limited. Due to decrease in tissue mass as well as muscle weakness, lung capacity declines with age even in healthy individuals (Tolep *et al*., 1995; Polkey *et al*., 1997). As senescence progresses, lung tissue becomes prone to inflammation, fibrosis and tumors demolishing lung capacity. Infections are frequent in the pulmonary tract of the elderly, leading to a chronic cycle of injury and repair that causes significant changes in the structure, function and gene expression of alveolar epithelial cells contributing to the development of chronic pulmonary diseases (Baarsma *et al*., 2011; Chilosi *et al*., 2012). Studies suggest that the senile lung is characterized by airspace enlargement similar to acquired emphysema (Verbeken *et al*., 1992) even detected in nonsmokers above 50 years of age (Sharma & Goodwin, 2006; Calvi *et al*., 2011). Similarly to humans, aging of the mouse lung is associated with homogeneous airspace enlargement.

The aging process of the lung is complex both in test animals and humans. Apart from decreased ability to withstand infections, low level chronic inflammatory processes are frequently detected (Meyer *et al*., 1996). The low level chronic inflammation is associated with tissue destruction requiring effective tissue regeneration (Crosby & Waters, 2010) coordinated by epithelial progenitor cells. The progenitor cells originate from the five putative stem cell niches primarily identified in the lungs of mice (Engelhardt, 2001). The cells responsible for cellular regeneration in the bronchiolar region are the nonciliated epithelial cuboid Clara cells (Park *et al*., 2006) while in the gas-exchange region of the alveoli, ATII cells drive the regenerative process. ATII cells are capable of transdifferentiation into ATI cells (Crosby & Waters, 2010; Rock *et al*., 2011; Barkauskas *et al*., 2013) providing the gas exchange surface of alveoli. ATII cells are also important in producing surfactant proteins responsible for lowering surface tension in the alveoli aiding gas exchange and stabilizing alveolar structure (Rooney *et al*., 1994) Surfactants also have immune-modulatory activity in the host defense system (Veldhuizen & Possmayer, 2004; Maina *et al*., 2010) making the presence of a well-maintained ATII cell population essential.

Although ATII cells are vitally important, ATII-s are unable to take up triglycerides directly from the blood and need the help of lipofibroblasts (Torday *et al*., 1995; Rehan & Torday, 2012). Lipofibroblasts can take up triglycerides and accumulate lipid droplets generated by a proliferator-activated receptor gamma (PPARγ) (Ferguson *et al*., 2009) and adipose differentiation-related protein (ADRP) (Gao & Serrero, 1999; Schultz *et al*., 2002)-dependent mechanism.

In recent studies, the secreted Wnt glycolipoprotein ligand family (Pongracz & Stockley, 2006) have been reported to regulate both aging (Brack *et al*., 2007) and PPARγ activity (Takada *et al*., 2009; Talaber *et al*., 2011). The two main and best characterized Wnt pathways are the β-catenin-dependent or canonical and the β-catenin-independent or noncanonical pathways (Pongracz & Stockley, 2006). While the canonical Wnts antagonize the PPARγ function (Takada *et al*., 2009), noncanonical Wnts have not been reported to affect PPARγ transcription or activity. Recent studies conducted in aging mice have connected PPARγ to lipofibroblast differentiation (Willis & Borok, 2007; Paxson *et al*., 2011). If PPARγ is reduced, lipofibroblasts differentiate into myofibroblast not supporting the maintenance of ‘stemness’ or surfactant synthesis (Königshoff & Eickelberg, 2010; Paxson *et al*., 2011).

Based on the above data, we theorized that a well-balanced Wnt microenvironment is essential in the alveolar epithelial maintenance mechanism. To investigate the stability of the Wnt microenvironment and the availability of lipofibroblasts in the aging lung, studies were performed using Balb/c mice, primary human lung tissues as well a three-dimensional (3D) human lung tissue model. Our results demonstrate that increased expression of Wnt4 leads to down-regulation of PPARγ in fibroblasts and epithelials cells increasing myofibroblast and inhibiting lipofibroblast differentiation, resulting in decreased surfactant production.

## Results

### Morphological changes in the aging lung

During aging, pulmonary function deteriorates due to pulmonary inflammation and structural changes described as senile emphysema. Microcomputed tomography (Fig. S1A,B, Supporting information) and hematoxilyn–eosin staining (Fig. S1C,D, Supporting information) of lung sections confirm such changes in Balb/c mice. Both techniques highlighted enlarged airspace in the distal lung regularly coinciding with decreased gas-exchange surface. Similar airspace enlargement has also been observed in old human lungs measured by a 2.5-fold increase in mean alveolar diameter (Fig. S1E,F, Supporting information).

**Figure 1:**
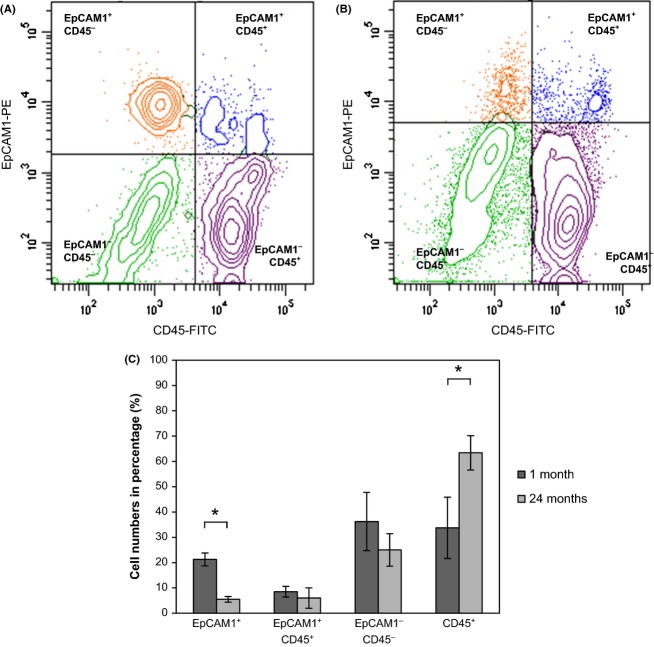
Flow cytometric analysis of cell populations in (A): 1-month and (B): 24-month lungs of Balb/c mice (EpCAM1^+^, CD45^+^, EpCAM1^-^CD45^-^, EpCAM1^+^CD45^+^) (C): Bar chart of young and old mouse lung cells. The numbers of different cell subsets are shown in percentage (*P < 0.05).

To confirm degeneration of the epithelial surface layer during aging, single cell suspensions were generated from the pulmonary tissues of mice, and cells were sorted based on EpCAM1 and CD45 cell surface antigens. Analysis of density plots (Fig. 1A,B) revealed a significant increase in CD45+ leukocyte level showing a marked increase in both macrophage and B-cell populations (Fig. S1G, Supporting information), whereas the number of epithelial cells significantly decreased in the senescent lung (Fig. 1C).

### Both PPARγ expression and lipid levels decrease with age

**Figure 2:**
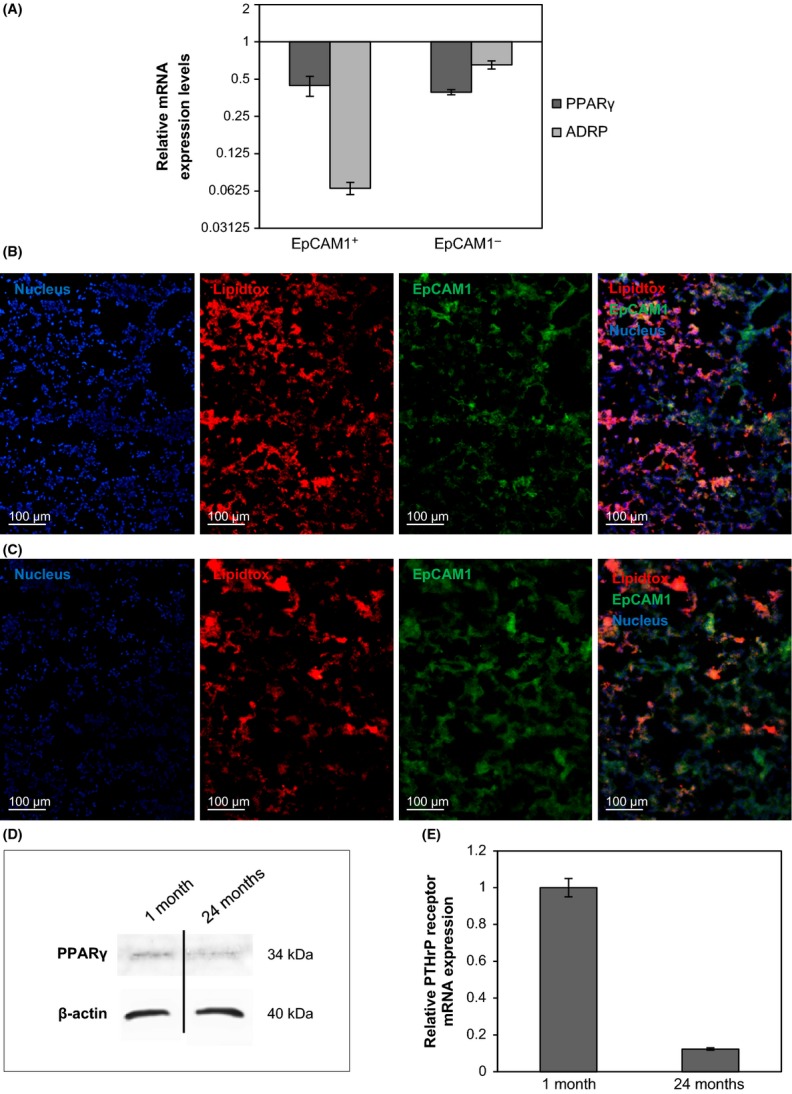
(A): mRNA expression levels of adipose differentiation markers in 24-month-old Balb/c mouse lung epithelial and nonepithelial cells, compared with 1 month-old. Both the PPARγ and the ADRP are decreased at 24 month-old compared with the 1-month-old lung cell types. LipidTox staining of (B): 1-month-old and (C): 24-month-old Balb/c mouse lung sections showing nuclei staining, lipid staining (LipidTox), and EpCAM1-FITC staining individually then in a merged picture. (D): PPARγ protein levels were detected in 1-month-old and 24-month-old lung extracts by Western blotting. Equal protein loading was tested using anti-β-actin antibody. The blot is a representative of two individual experiments. (E): PTHrP receptor mRNA levels were measured in EpCAM-/CD45- cell populations of 1-month-old and 24-month-old Balb/c mouse lungs using qRT–PCR analysis. (The graph is a representative of three individual experiments).

While senescence-associated low level chronic inflammation explains tissue destruction, the reasons for ineffective regeneration are not so easily explained. As lipofibroblasts are essential for maintenance of ATII-s, molecular studies were designed to identify molecules involved in lipid production to define the presence and activity of lipofibroblasts. PPARγ mRNA as well as its downstream target, adipose differentiation-related protein (ADRP), were measured in purified EpCAM1^+^ epithelial and EpCAM1^−^ nonepithelial cells using qRT–PCR (Fig. 2A). Compared with 1-month-old Balb/c mice, both PPARγ and ADRP levels decreased at 24 months of age in both cell populations indicating reduced ability for lipid synthesis in the aging lung. To confirm the qRT–PCR data, lipid levels were assessed in pulmonary tissues using neutral lipid staining of 1-month-old and in 24-month-old mouse lung sections (Fig. 2B,C). While lipid staining co-localized with nuclear staining in young (1-month-old) mice (Fig. 2B), lungs of old (24-month-old) mice contained enlarged lipid droplets not associated with nuclear staining (Fig. 2C). Western blots were performed using protein extracts of 1-month- and 24-month-old mouse lungs (Fig. 2D). The results show an age-associated reduction in PPARγ protein levels indicating a loss of lipofibroblasts. According to Torday *et al*. (Torday *et al*., 2003), parathyroid hormone-related protein (PTHrP) expression is necessary for differentiation of mesenchymal lipofibroblasts, which induce ATII cell differentiation making PTHrP receptor transcript levels indicative of alterations in lipofibroblast differentiation. qRT–PCR analysis revealed a drastic reduction in PTHrP receptor mRNA levels within the EpCAM1^-^/CD45^-^ cell population (Fig. 2E) indicating loss of lipofibroblasts in the aging lung.

### Alteration of the Wnt microenvironment during pulmonary senescence

Recent studies have highlighted the importance of Wnt signaling in the regulation of PPARγ activity. Takada reviewed (Takada *et al*., 2009) that canonical Wnts antagonize the effect of PPARγ in osteoblast–adipocyte differentiation. Moreover, Talabér *et al*. (Talaber *et al*., 2011) have shown that overexpression of Wnt4 in TEP1 thymic epithelial cell line results in reduced PPARγ expression and consequent inhibition of thymic adipose involution.

**Figure 3:**
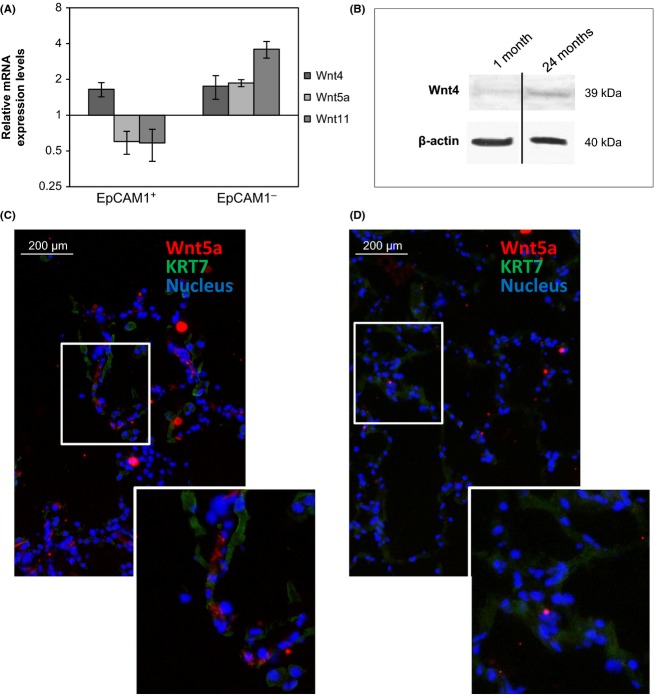
(A): mRNA expression levels of different Wnt molecules in 24-month-old Balb/c mouse lung epithelial and nonepithelial cells were measured using qRT–PCR analysis and relative expression was determined to β-actin, then compared with Wnt expression in 1-month-old test animals. (B): Wnt4 protein levels were detected in 1-month-old and 24-month-old lung extracts by Western blotting. Equal protein loading was tested using anti-β-actin antibody. (The blot is a representative of two individual experiments). Immunofluorescent staining of Wnt5a in (C): 73-year and (D): 21-year human lung sections (The staining is representative of three separate experiments).

Several Wnts, including Wnt4 as well as the inflammatory mediator, Wnt5a were measured in both epithelial and nonepithelial cells of mouse lungs. While Wnt4 mRNA levels increased (Fig. 3A) during aging in both epithelial (EpCAM1^+^) and nonepithelial (EpCAM1^−^) cells, both Wnt5a and Wnt11 expression decreased in epithelial (EpCAM1^+^) and increased in nonepithelial (EpCAM1^−^) (Fig. 3A) cells during senescence. Western blot analysis of protein extracts of 1-month- and 24-month-old mouse lungs supported the age-associated increase in Wnt4 levels (Fig. 3B). Wnt5a staining of 73-year-old (Fig. 3C) and 21-year-old (Fig. 3D) human lungs reinforced similarities between the mouse and human pulmonary senescence program. Corresponding to mouse qRT–PCR data, Wnt5a-staining intensity increased with age in the human lung and Wnt5a was detected in the nonepithelial, cytokeratin-7 negative cell population (Fig. 3C).

### β-catenin-dependent regulation of PPARγ expression

**Figure 4:**
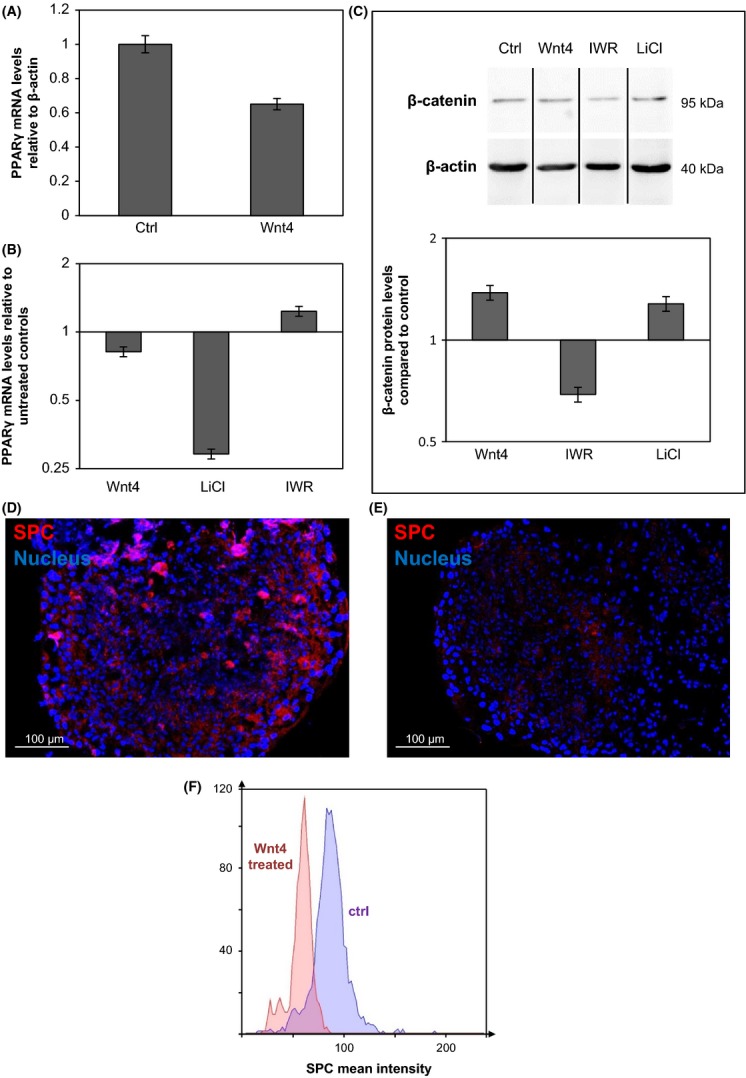
(A): PPARγ expression levels in 3D human lung tissue models, following 7-day exposure to control (ctrl) and Wnt4 supernatants of thymic epithelial cells (TEP1). PPARγ mRNA expression levels were determined by qRT–PCR analysis following 7-day exposure to control (ctrl) and Wnt4-enriched supernatants of TEP1, to 10 mm LiCl and to 1 μm IWR in (B): primary human lung fibroblast (NHLF) cells. (C): β-catenin protein levels were determined in NHLF cells after exposure to Wnt4-enriched supernatants of TEP1, 10 mm LiCl and 1 μm IWR for 7 days. Equal protein loading was determined using anti-β-actin antibody. (The blot is a representative of two individual experiments). The blots were then densitometrically scanned and plotted against the controls. Pro-SPC staining on (D): ctrl and E: Wnt4-enriched supernatant treated 3D human lung tissue model (red: pro-SPC, blue: DAPI stained nuclei) (The staining is representative of three separate experiments). F: Mean intensity differences in pro-SPC staining in Wnt4-treated and Ctrl 3D human lung tissue models. (The graph is a representative of three individual experiments).

Our previous data (Talaber *et al*., 2011) as well as Takada’s results (Takada *et al*., 2009) designated the canonical Wnt signaling pathway as regulator of PPARγ expression. As Wnt4 but not Wnt5a or Wnt11 can act via the canonical Wnt signaling pathway (Pongracz & Stockley, 2006), our attention was focused on Wnt4. First 3D human lung tissue models were exposed to control and Wnt4 supernatants of TEP1 cells for 7 days. Using qRT–PCR, reduced mRNA expression levels of PPARγ were measured (Fig. 4A). Thereafter, canonical Wnt pathway activity was modified using chemical activators and inhibitors of the β-catenin pathway. LiCl was used as activator inhibiting the activity of glycogen-synthase kinase (GSK)3-β, which enzyme phosphorylates and labels β-catenin for proteosomal degradation. IWR is an inhibitor stabilizing Axin proteins that play a role in β-catenin destruction (Pongracz & Stockley, 2006).

Primary human lung fibroblasts (NHLF) were treated with LiCl at 10 mm, IWR at 1 μm concentration and with Wnt4-enriched supernatant of TEP1 cells for 7 days, then PPARγ mRNA levels were measured using qRT–PCR. The expression of PPARγ was drastically reduced after LiCl and Wnt4 treatment (Fig. 4A,B), while inhibition of β-catenin signaling increased PPARγ levels. β-catenin levels were also assessed by Western blot analysis after treatment of NHLF cells with LiCl, Wnt4 and IWR. While LiCl and Wnt4 treatment increased, IWR treatment reduced β-catenin protein levels (Fig. 4C) indicating that amplified Wnt4 production during aging is likely to initiate reduction of lipofibroblast differentiation via a β-catenin-dependent mechanism. To examine the possibility that Wnt4-induced reduction of PPARγ levels affect SPC production, a distal human lung tissue model was set up using small airways epithelial cells (SAEC) and NHLF in three-dimensional (3D) human lung tissue models (Fig. S2, Supporting information). As the 3D human lung tissue model showed reduced inflammatory cytokine and increased differentiation marker expression both at levels of pro-SPC and Aquaporins (Fig. S2, Supporting information), it was determined to be a suitable model system for molecular analysis of surfactant regulation. The 3D lung tissue model was treated with Wnt4-enriched supernatants and rhWnt5a, respectively. Following 7 days of incubation, Wnt4 reduced pro-SPC expression, demonstrating that Wnt4 can regulate pro-SPC levels (Fig. 4D–F). In contrast, rhWnt5a treatment had no significant effect on pro-SPC expression (Fig. S4A,B), supporting the theory that Wnt4 is the likely regulator of PPARγ and surfactant expression.

### Reduced β-catenin activity is necessary in pulmonary epithelial cells to produce pro-SPC

**Figure 5:**
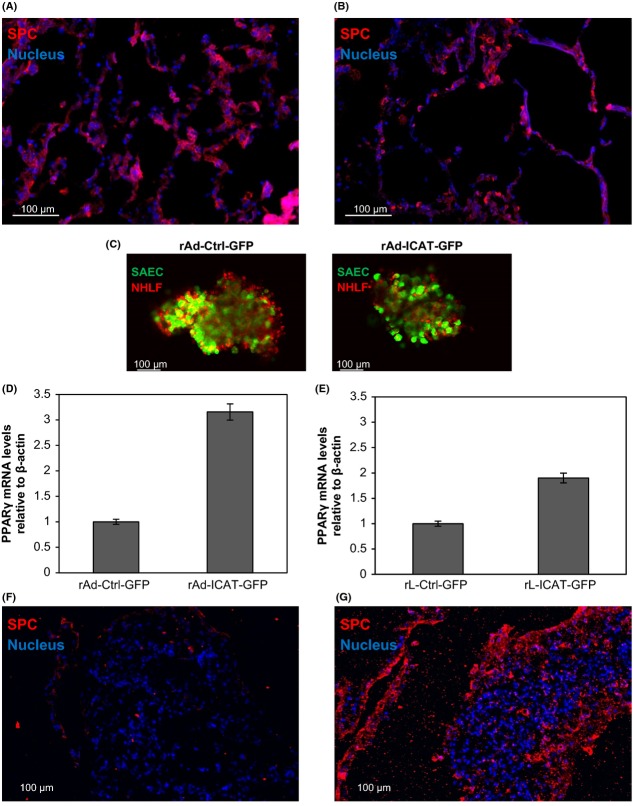
Immunofluorescent staining of pro-surfactant protein C (pro-SPC) in (A): 21-year-old and (B): 73-years-old human lung tissue sections. (C): Confocal picture of intact 3D human lung tissue models infected with rAd-Ctrl-GFP and rAd-ICAT-GFP. SAEC are expressing GFP (green) within the 3D human lung tissue model. NHLF cells were prestained with Dil (red). (D): PPARγ mRNA expression levels were determined by qRT–PCR analysis in 3D human lung tissue model following 7-day suppression of β-catenin activity by ICAT specifically within the SAEC population using rAd gene delivery. (E): PPARγ mRNA expression levels were determined by qRT–PCR analysis in 3D human lung tissue model following suppression of β-catenin activity by ICAT specifically within the NHLF cell population using rL gene delivery. Immunofluorescent staining of pro-SPC in (F): control 3D human lung tissue model containing rAd-Ctrl-GFP SAEC and (G): ICAT overexpressing 3D human lung tissue model containing rAd-ICAT-GFP SAEC (the staining is representative of three separate experiments).

While the above data support that PPARγ levels in fibroblasts are necessary for lipofibroblast-like differentiation and maintenance of surfactant production in ATII-type cells, it is still not clear whether PPARγ activity is needed within the ATII cell population for surfactant synthesis. It is an important question as age-associated decline of PPARγ mRNA affected not only in fibroblasts but epithelial cells also (Fig. 2A). As the aging process in the human lungs was associated with reduced pro-SPC levels (Fig. 5A,B), the presence and activity of PPARγ in pulmonary epithelium might be equally important to that in lipofibroblasts.

β-catenin activity was modulated therefore using the physiological inhibitor of the β-catenin-dependent Wnt signaling pathway, ICAT. ICAT (Graham *et al*., 2002) was introduced into epithelial as well as fibroblast cells using recombinant viral gene delivery methods in the 3D human lung tissue model. To specifically target epithelial cells (SAEC), recombinant Adeno viruses (rAd-GFP and rAd-ICAT-GFP) were used (Fig. 5C), while fibroblasts (NHLF) were transfected using lentiviruses (rL-GFP and rL-ICAT-GFP). For lentiviral gene delivery, NHLF cells were infected before the generation of the 3D human lung tissue model to avoid transfection of epithelial components. Following 7 days of exposure to ICAT, PPARγ expression was measured. Inhibition of β-catenin activity either in epithelium or in fibroblasts drastically increased PPARγ expression (Fig. 5D,E), indicating that inhibition of β-catenin signaling modulates lipid metabolism of both cell types. Pro-SPC-staining increased drastically in rAd-ICAT-GFP-infected tissues (Fig. 5F,G) identifying a β-catenin-regulated and PPARγ-dependent mechanism as an important element of pro-SPC production in ATII cells.

### Wnt signaling in myofibroblast-like differentiation

**Figure 6:**
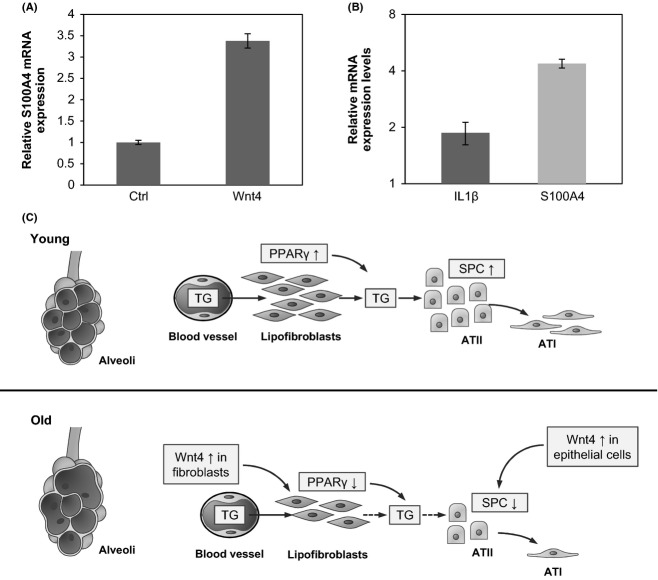
(A): Relative gene expression levels of S100A4 in Wnt4-enriched supernatant treated human lung tissue spheroids. qRT–PCR analysis of gene expression is presented as relative to controls (data is representative of two separate experiments). (B): Relative gene expression levels of IL1β and S100A4 in rhWnt5a-treated 3D human lung tissue models. qRT–PCR analysis of gene expression is presented as relative to untreated controls (data are representative of three separate experiments). (C): Schematic summary of molecular changes in the distal lung during senescence (TG: triglyceride; SPC: pro-surfactant protein C, PPAR: peroxisome proliferator-activated receptor gamma).

According to Torday *et al*. (Torday *et al*., 2003), transdifferentiation of lipofibroblasts to myofibroblasts is characterized by loss of PTHrP receptor expression and triglyceride content. Our results support previous findings as PTHrP mRNA levels decreased with age in the nonepithelial cell population (Fig. 2E). To investigate whether changes in the Wnt microenvironment are responsible for increased myofibroblast differentiation, the myofibroblast marker S100A4 mRNA was measured after exposure of NHLF cells and the 3D human lung tissue model to Wnt4 and Wnt5a, respectively. Interestingly, both Wnt4 and Wnt5a were able to increase S100A4 transcript levels (Fig. 6A,B), indicating involvement in myofibroblast-differentiation.

## Discussion

During senescence, changes in the structure and cellular composition of the lung leads to airspace enlargement that occur even in nonsmoker, healthy adults (Tolep *et al*., 1995). Investigating the molecular background of the pulmonary aging process has highlighted that both in test animals and in humans the structural differences are triggered by very similar molecular alterations in Wnt signaling. Up-regulation of Wnt4 in epithelial and nonepithelial cells as well as up-regulation of Wnt5a in nonepithelial tissue compartments can lead to far reaching consequences.

Pinpointing the initial molecular trigger, however, is difficult. Increased Wnt5a levels have been reported to regulate accumulation of leukocytes and increased inflammatory cytokine production (Li *et al*., 2011; Briolay *et al*., 2013) as well as senescence (Florian *et al*., 2013) in many tissues. Previous studies have also described Wnt5a to increase fibroblast proliferation (Vuga *et al*., 2009), increased inflammatory cytokine production (Rauner *et al*., 2012) and epithelial cell migration (Bartis *et al*., 2013) providing further insight into the molecular environment favoring fibrosis and cancer development in the elderly lung.

Increased Wnt5a secretion of fibroblast cells of the aging lung can stimulate inflammation and therefore maintenance of a damaging environment to the gas-exchange surface. If the damaging microenvironment is prolonged and coincides with a reduced regenerative capacity, the outcome can be fatal. Decreased number of the alveolar progenitor or ATII cells (Torday *et al*., 2003; Yee *et al*., 2006) could explain such age-associated reduction in regeneration. ATII cells serve not just as alveolar stem cells, but they are also the source of surfactants. Reduction in pro-SPC levels indicates weakened functionality of ATII-s that was documented in both aging test animals as well as in primary human lung tissue.

Our studies have shown that alterations in Wnt secretion and consequent modulation of intracellular signaling interferes with the lipid metabolism at PPARγ level. The Wnt4-induced reduction in PPARγ levels seems especially important as PPARγ and its down-stream target, ADRP are responsible for the uptake of triglycerides from the blood stream (Varisco *et al*., 2012) that is essential for surfactant production in ATII cells. Our data have also shown that PPARγ is necessary in epithelial cells to regulate surfactant production. As surfactant lining of the alveoli is essential to uniform inflation and to protect against chemical or particulate injury of the distal lungs, a decrease in surfactant production can accelerate and maintain lung tissue damage and speed up aging.

Up-regulation of Wnt4 and Wnt5a has an additional impact on aging lung tissue. Both Wnt4 and Wnt5a can increase myofibroblast (S100A4) marker expression, indicating that ATII cells are left without their essential support mechanism (lipofibroblast) (Torday *et al*., 2003; Rehan & Torday, 2012) during senescence. Additionally, Wnt-induced myofibroblast differentiation might also be linked to modulation of tissue damage control, as when tissue or parenchymal repair is needed myofibroblasts function as elemental emergency cells.

While modification of Wnt expression in absolute levels are relatively little, the distal lung seems unable to adapt to shifts in the molecular microenvironment (Fig. 6C). Especially, as changes taking place simultaneously are magnified at several levels. An increase in Wnt4 expression leads to PPARγ down-regulation that paralyses lipofibroblast differentiation and quite possibly reduces the ability of ATII cells for surfactant production. As in the absence of lipofibroblasts, ATII-s can no longer replenish their triglyceride sources from the blood, surfactant production can become drastically reduced. In the absence of surfactants, the lung tissue becomes prone to infections (LeVine *et al*., 2002; Kazi *et al*., 2010). During the course of infection, alveolar epithelial cells start to produce Wnt5a that adds to the already increased Wnt5a levels produced by the aging nonepithelial elements of the distal lung. The simultaneous increase in inflammatory cytokines produced by the infiltrating immune cells that attack the site of infection can increase Wnt5a levels even further (Rauner *et al*., 2012). Meanwhile, the damaged alveolar epithelial lining is unable to regenerate as the number of functional ATII cells is decreasing leaving the damaged tissue in need of myofibroblasts to close the wound.

Further studies are necessary and are underway to investigate the above described mechanism *in vivo* and to explore the potential clinical applications of the pulmonary repair mechanism.

## Experimental procedures

### Ethical statement

Lung tissue samples were collected during lung resections at the Department of Surgery, University of Pécs, Hungary. The project was approved by the Ethical Committee of the University of Pécs. Patients had given written consent to provide samples for research purposes. All collected samples were treated anonymously.

### Animals

For the experiments Balb/C inbred, albino mice were used from both genders. The mice were kept under standardized conditions, where tap water and food was provided ad libitum.

### Sky scan microCT

Mice anesthetized intraperitoneally with sodium pentobarbitol (eutazol) were placed in the skyscan 1176 microCT (Bruker, Kontich, Belgium) machine equipped with a large format 11 megapixel camera. The pictures were taken from the lung in 180°. The reconstitution was performed with skyscan software, which integrates a physiological monitoring subsystem providing breathing and heartbeat gating for thoracic image improvement through synchronized acquisition. Then the pictures were calibrated with ctan software (Bruker). The 3D pictures were generated from the calibrated images by CtVox (Bruker). Colors were based on Hounsfield scale, where red was placed at −1000 and represents free air, and through tones, blue represents lung tissue.

### Lung cell isolation

Mice were anesthetized with 1% of sodium pentobarbital through intraperitoneal injection (70 μL/10 g). Then abdominal aorta was intersected, and mice were perfused through right ventricle with 10 mL of phosphate-buffered saline (PBS) to reduce lung blood content. 3 mL trypsine (%) to initiate the fine digestion, 10 mL PBS to wash out trypsine, 3 mL collagenase-dispase (3 mg mL^−1^ collagenase (Sigma-Aldrich, St. Louis, MO, USA) 1 mg mL^−1^ dispase (Roche F. Hoffmann-La Roche Ltd. Basel, Switzerland) 1u μL^−1^ DNAse I (Sigma-Aldrich). Finally, the lung was filled up with the collagenase-dispase solution trough the trachea. Lungs were removed from the chest and separated from the heart and thymus and cleaned from connective tissue. Pulmonary lobes were dissected into smaller pieces and digested in 10 mL collagenase-dispase for 50 min with continuous stirring. Digested lung cells were filtered with 70-μm cell-strainer (BD Becton, Dickinson and Company Franklin Lakes, NJ, USA)

### Cell sorting

Single cell suspension isolated from mouse lungs were labeled with anti-CD45-FITC produced at the University of Pécs, Department of Immunology and Biotechnology (Balogh *et al*., 1992) and anti-EpCAM1 (G8.8 anti-rat-PE). Cell sorting was performed by FACSAria III (BectonDickinson) cell sorter. The following populations were collected: EpCAM^-^CD45^-^ EpCAM^+^CD45^-^ EpCAM^+^CD45^+^ and EpCAM^+^CD45^-^. The purity of sorting was above 99%.

### Cell lines

During our experiments, normal human lung fibroblast (NHLF) cells were exposed to TEP1 Wnt4 overexpressing supernatant and to normal TEP1 cell normal supernatant as control (Talaber *et al*., 2011) for 7 days. Fibroblast cells (NHLF) were also treated with the β-catenin pathway activator LiCl at the concentration of 10 mm for 7 days and the β -catenin pathway inhibitor, IWR at 1 μm (Sigma) for two days. After RNA isolation and cDNA synthesis, PPARγ levels were measured by qRT–PCR using PPARγ- and β-actin-specific primers.

### Three-dimensional (3D) human lung tissue model

Primary small airway epithelial cells (SAEC) and normal human lung fibroblasts (NHLF) cells were purchased from Lonza (Walkersville, MD, USA). All cell types were isolated from the lungs of multiple random donors of different sexes and ages. We used Small Airway Epithelial Growth medium (SAGM) or fibroblast growth medium (FGM) for the initial expansion of SAEC or NHLF respectively, as recommended by the manufacturer (Lonza). All types of cell cultures were incubated in an atmosphere containing 5% CO2, at 37 °C. For 3D culturing, cells were mixed at 1:1 ratio, dispensed onto V-bottom 96-well plates (Sarstedt Nümbrecht, Germany) and were immediately pelletted at 600xg for 10 min at room temperature. Then spheroids were kept in 24-well plate (Sarstedt) in mixed SAGM:FGM (1:1 ratio) medium. They were exposed to rhWnt5a and Wnt4 supernatant for 7 days.

### Recombinant Adeno (rAd) and Lenti (L) viral constructs and rAd and L-viral infecton of pulmonary epithelium (SAEC) and fibroblasts (NHLF)

ICAT sequence was amplified by PCR using forward (5′) 5′-ATGAACCGCGAGGAGCA-3′ and reverse (3′) 5′-CTACTGCCTCCGGTCTTCC-3′ primer sequences and cloned into the bicistronic GFP (green fluorescence protein) Adeno Shuttle and Lenti pWPTS vectors. The Shuttle vector was cloned by homologous recombination into the adenoviral vector. Adenovirus was produced by transfecting the linearized plasmid DNA into the 293 packaging cell line (American Type Culture Collection, Rockville, MD, USA) using Lipofectamine 2000 (Invitrogen, Carlsbad, CA, USA). The resulting plaques were amplified, the adenovirus purified and concentrated using the adenoviral purification kit (BD Biosciences, Franklin Lakes, NJ, USA).

Late second-generation lentiviral vectors were prepared by co-transfection of three plasmid constructs (envelope construct pMD.G, packaging construct R8.91 and transfer construct pWPTS) into 293T cells using the calcium phosphate method as described previously (Bovia *et al*., 2003). Biological titration was performed with HeLa cells. Viral particles were concentrated 1000-fold in volume; biological titers reached 10^8^ TU mL^−1^. The HIV-1-derived lentiviral system was kindly provided by Prof. Didier Trono (CMU, Geneva, Switzerland).

For ICAT delivery to epithelial cells, complete SAEC-NHLF spheroids were incubated in the rAd virus containing media for 1 h, then the SAEC-(ICAT-GFP)-NHLF and SAEC-(GFP)-NHLF shpereoids were washed and incubated for 7 days before RNA isolation. For ICAT delivery to fibroblasts, NHLF cells were exposed to L-virus containing media for 1 h, then the cells were washed and incubated for 2 days in 2D monocultures. NHLF cells were then harvested, and spheroids were produced as described above. SAEC-NHLF-(ICAT-GFP) and SAEC-NHLF-(GFP) shperoids were cultered for an additional 5 days before RNA isolation.

### Western blot analysis

Lung tissues of young (1 months) and old (24 months) lyzed in lysis buffer (20 mm HEPES pH 7.4, 1 mm MgCl_2_, 1 mm CaCl_2_ 137 mm NaCl, 50 mm β-glycerophosphate, 2 mm EGTA, 1% Triton ×100 supplemented with 1 mm DTT, 2 mm PMSF, 2 μg mL^−1^ leupeptin, 1 μg mL^−1^ aprotinin, 1 μg mL^−1^ pepstatine) on ice for 20 min, then snap-frozen in liquid nitrogen and stored at −70 °C until used. Just before loaded on 10% SDS-PAGE, the samples were boiled in 2× SDS sample buffer. Protein concentrations of lung extracts were measured with bicinchoninic-acid kit (Sigma), then the same amount of proteins were loaded to polyacrylamide gels then transferred to nitrocellulose membrane. The membrane was blocked with TBS buffer containing 1% of BSA and 0.05% of Tween and incubated with primary antibodies (anti-β-catenin, anti-Wnt4 both purchased from Santa Cruz, both at 1:1000 dilution) overnight. HRP-conjugated anti-Mouse (Sigma) and anti-Goat (Sigma) were used as secondary antibodies (both at 1:1000 dilution). Blots were visualized using the chemiluminescent Supersignal kit (Pierce) and densitometrically scanned for quantification (LAS 4000).

### RNA isolation

Cell samples were homogenized in RA1 reagent, and RNA was isolated using the NucleospinII RNA isolation kit (Macherey-Nagel, Dueren, Germany). DNA digestion was performed on column with RNase-free DNase. The concentration of RNA samples was measured using Nanodrop (Thermo Scientific, Waltham, MA, USA).

### Real-Time quantitative PCR

cDNA was synthesized with high-capacity RNA to cDNA kit (Life technologies Inc., Carlsbad, CA, USA) using 1 μg of total RNA according to manufacturer’s recommendation. Reverse transcription was performed in 20 μL total volume using random hexamer primers. RT–PCR was used for gene expression analysis. Gene expression levels were determined by gene-specific RT–PCR using ABsolute QPCR SYBR Green Low ROX master mix (ABGene, Thermo Scientific) and 100 nm primers on the Applied Biosystems 7500 thermal cycler system. For normalization, β-actin was used as housekeeping gene. The primer sequences are shown in the Table 1. PCR conditions were set as follows: one cycle 95 °C for 15 min, 40 cycles 95 °C for 15 s, annealing temperature was 58 °C and 72 °C for 1 min for elongation. Specification of the PCR was determined by using a dissociation stage. The calculation of the RT–PCR results was performed as follows: The mean Ct values are determined by calculating the average of the parallel samples. ΔCt is calculated by subtracting the mean Ct of the housekeeping gene from the mean Ct of the gene of interest. ΔΔCt is constituted by the difference between the old sample Ct and the young sample as a control Ct values. Finally, the relative quantity (RQ), which is presented in the diagrams, can be calculated by applying the formula: RQ = 2^–ΔΔCt.^

**Table 1:** Primer sequences

Primers	Forward	Reverse
Mouse β-Actin	TGGCGCTTTTGACTCAGGA	GGGAGGGTGAGGGACTTCC
Mouse PTHrPR	GGCGAGGTACAAGCTGAGAT	ACACTTGTGTGGGACACCAT
Mouse Wnt4	CTCAAAGGCCTGATCCAGAG	TCACAGCCACACTTCTCCAG
Mouse Wnt5a	AAGCAGGCCGTAGGACAGTA	CGCCGCGCTATCATACTTCT
Mouse Wnt11	GCTCCATCCGCACCTGTT	CGCTCCACCACTCTGTCC
Mouse PPARγ	CCCAATGGTTGCTGATTACAAA	AATAATAAGGTGGAGATGCAGGTTCT
Mouse ADRP	CGCCATCGGACACTTCCTTA	GTGATGGCAGGCGACATCT
Human β-Actin	GCGCGGCTACAGCTTCA	CTTAATGTCACGCACGATTCC
Human PPARγ	GGTGGCCATCCGCATCT	GCTTTTGGCATACTCTGTGATCTC
Human S100A4	TGGAGAAGGCCCTGGATGT	CCCTCTTTGCCCGAGTACTTG
Human IL1β	TCAGCCAATCTTCATTGCTCAA	TGGCGAGCTCAGGTACTTCTG

### Immunofluorescence

Mice were anaesthetized with sodium pentobarbital intraperitoneally and then they were perfused through the right ventricle with PBS solution as described above. Then lungs were filled up with 1:1 ratio of PBS:cryostate embedding media (TissueTek Alphen an den Rijn, The Netherlands) and frozen down at −80 °C.

The human samples were kept in PBS containing 1% of FCS at room temperature till processing. The filling, freezing and sectioning steps were performed as described above.

At the endpoint of the treatment, the 3D human lung tissue models were carefully removed from the 24-well plates and embedded into TissueTek embedding media and immediately frozen down at −80 °C.

For histological observations, cryostat sections (7–9 μm) were fixed with cold acetone for 10 min.

### Antibodies, fluorescent imaging

Following the rehydration and blocking step (for 20 min in 5% BSA in PBS), immunofluorescent staining was performed. Anti-Wnt5a (Santa Cruz, Santa Cruz, CA, USA) antibodies were used as primary antibodies, and anti-EpCAM1-FITC (clone G8.8, American Type Culture Collection (ATCC) directly labeled antibody was used as a control staining for 1 h. For human samples, anti-pro-SPC antibody (Millipore Billerica, MA, USA) was used. For the spheroids, anti-KRT7 (DAKO, Agilent, Santa Clara, CA, USA) antibodies and anti-E-cadherin (AbCam, Cambridge, UK) antibodies were applied.

The secondary antibodies were northern light anti-mouse NL-493 and northern light anti-rabbit NL-557 (R&D systems, Minneapolis, MO, USA). The nuclei were counterstained with DAPI (Serva, Heidelberg, Germany). Pictures were captured using Olympus IX81 fluorescence microscope (Shinjuku, Tokyo, Japan) equipped with CCD camera and analysis software. Images were processed and analyzed with ImageJ.

Fluorescent images were analyzed, and the mean intensity was calculated with strataquest software (Biotech-Europe, Prague, Czech Republic).

### Hematoxylin–eosin staining

Preparation of mouse and human lung sections is described above. Following sectioning, the samples were immediately stained with hematoxylin and eosin. Pictures were scanned with Pannoramic Desk machine (3D Histech, Budapest, Hungary) then analyzed by Pannoramic viewer software (3D Histech).

### Neutral lipid staining

Mouse lung sections were made as described above. Cold acetone fixed sections were stained with anti-EpCAM1 antibody directly conjugated with FITC (ATCC clone G8.8), then the LipidTox (Life Technologies Inc.) staining was performed. Fluorescent images were captured and analyzed as described above.

### Statistical analysis

If applicable, data are presented as mean ± standard deviation (SD), and the effects between various experimental groups were compared with the Student *t*-test. *P* < 0.05 was considered as significant.

## Supporting Information

**Fig. S1** The structure of the lung during aging presented by SkyScan micro Computed Tomograph; photographs of lungs of (A): 1–month-old and (B): 24-month-old Balb/c mice were obtained in 180 degree and converted to 3D by CTVol software (Skyscan). **Fig. S2** Characterization of 3D lung micro-tissue model. **Fig. S3** Inflammatory cytokines in 2D vs 3D tissues. **Fig. S4** Pro-surfactant protein C expression following rhWnt5a treatment of the 3D human lung tissue model.
