# Supplementary figures and images for: Alteration in the Wnt microenvironment directly regulates molecular events leading to pulmonary senescence
Source: Aging Cell. 2014 Jul 1;13(5):838–49. doi: 10.1111/acel.12240 (PMC4331750; doi:10.1111/acel.12240)

**A**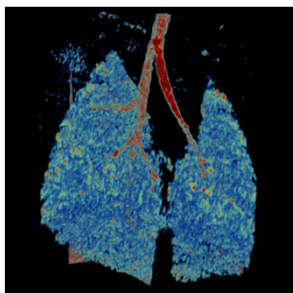**B**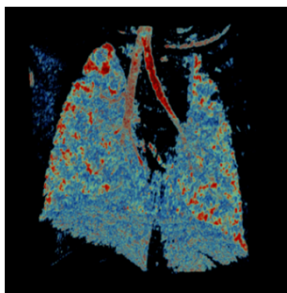**C**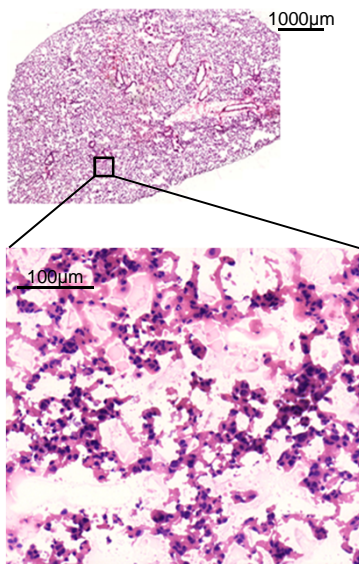**D**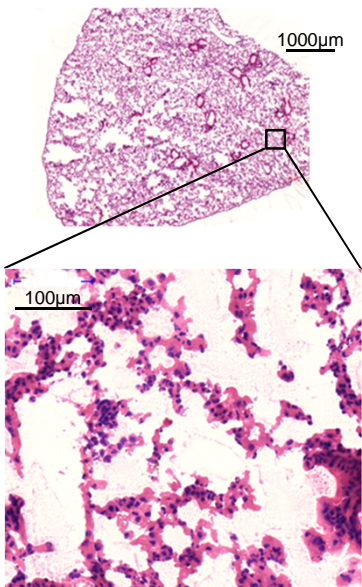**E**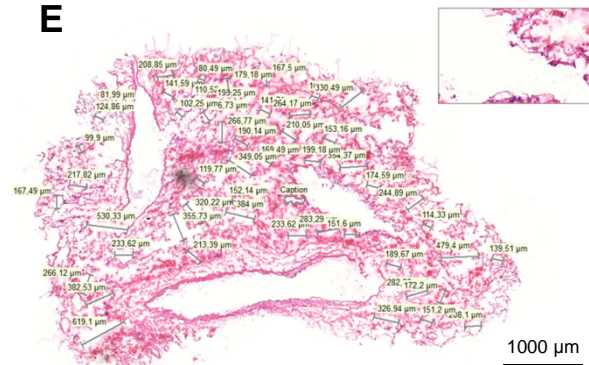**F**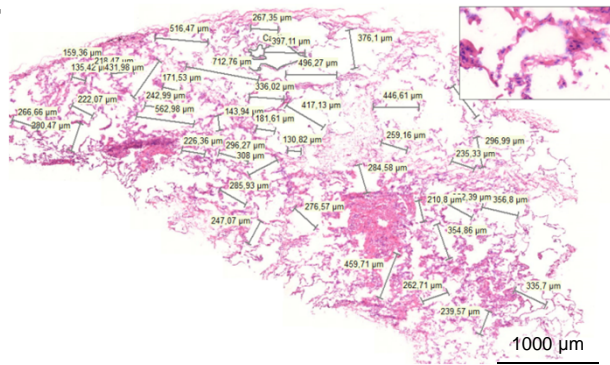**G**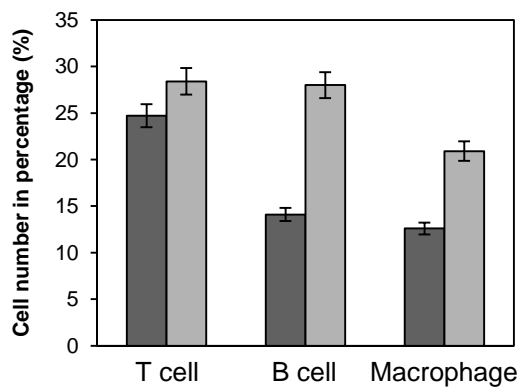

■ 1 month  
□ 24 months

**A**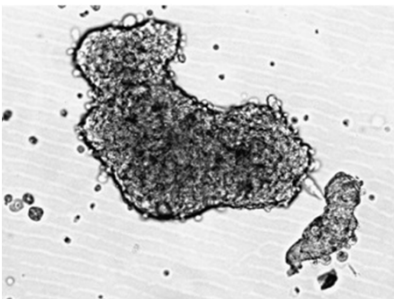**B**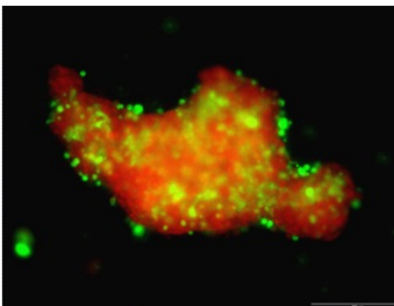**C**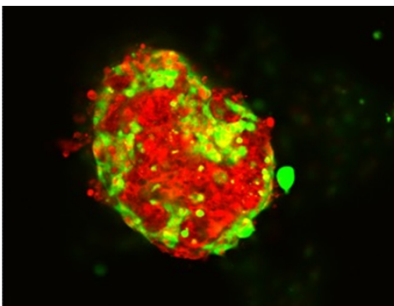**D**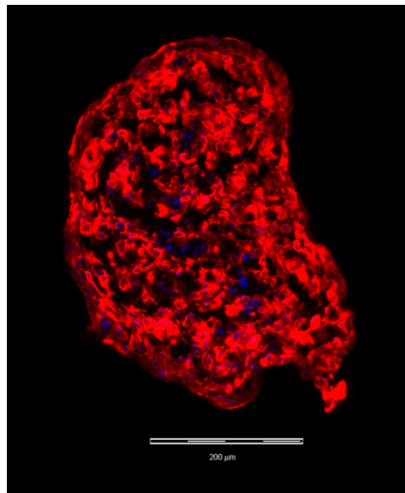**F**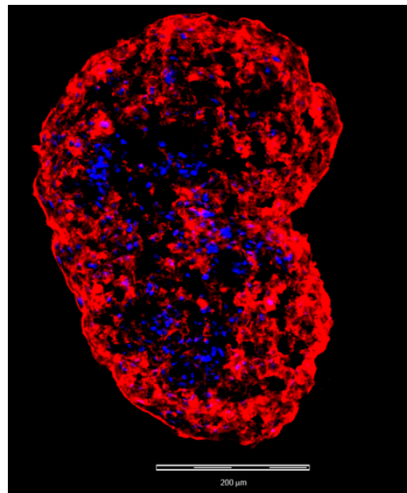**E**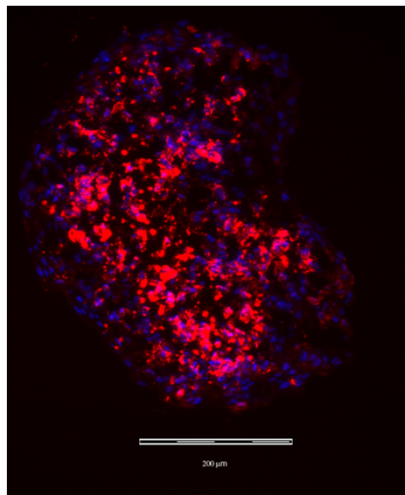**G**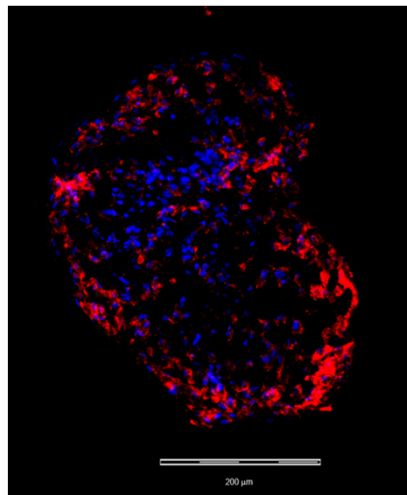**H**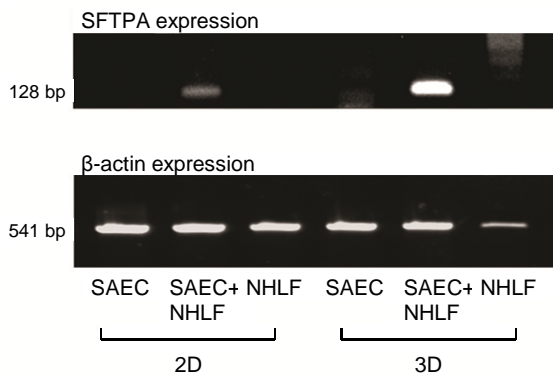**I**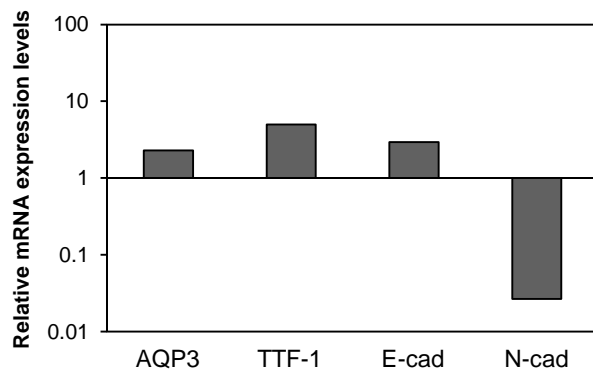

**A**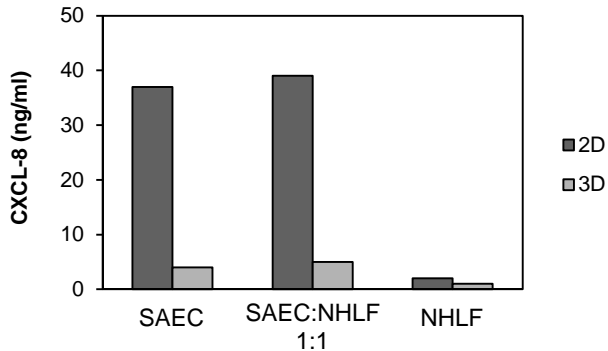**B**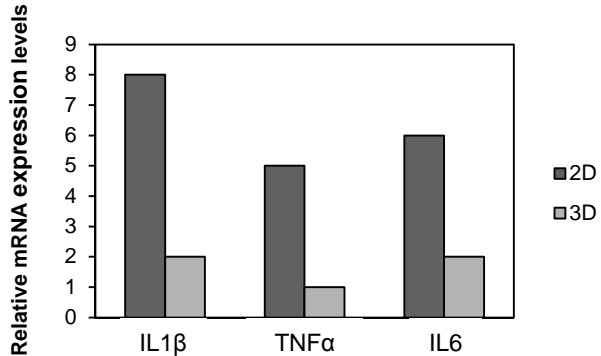

**A**

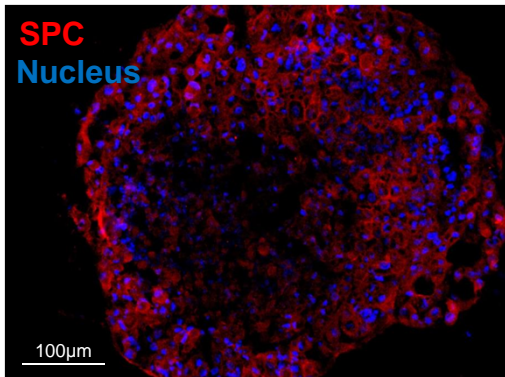

**B**

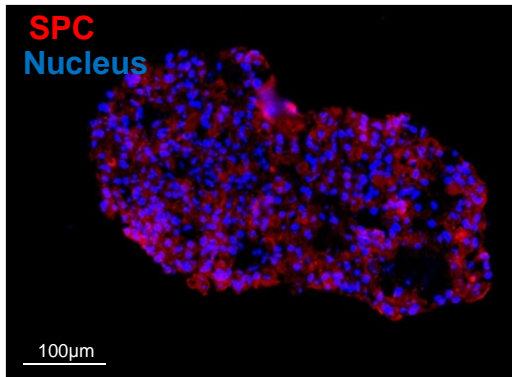

Supplement: Supplementary file 1 — Fig. S1 The structure of the lung during aging presented by SkyScan micro Computed Tomograph; photographs of lungs of (A): 1–month-old and (B): 24-month-old Balb/c mice were obtained in 180 degree and converted to 3D by CTVol software (Skyscan). Fig. S2 Characterization of 3D lung micro-tissue model. Fig. S3 Inflammatory cytokines in 2D vs 3D tissues. Fig. S4 Pro-surfactant protein C expression following rhWnt5a treatment of the 3D human lung tissue model. [file acel0013-0838-sd1.pdf]
